# Isolated Small Bowel Amyloidosis

**DOI:** 10.1093/jcag/gwab026

**Published:** 2021-08-13

**Authors:** Jenan Ghaith, Mariam Mukhtar, Talat Bessissow

**Affiliations:** Department of Medicine, University of Toronto, Toronto, Ontario, Canada; Division of Gastroenterology, Department of Medicine, King Abdulaziz University, Jeddah, Saudi Arabia; Division of Gastroenterology, Montreal General Hospital. McGill University Health Center, Montreal, Quebec, Canada

A 64-year-old male was assessed for incidental jejunal thickening noted on computed tomography (CT). The CT was performed for lower back pain resistant to analgesia. Medical history was notable for light chain amyloidosis in the left eye which was biopsied and resected eight years ago.

Upon further questioning, he reported a 2-year history of dull abdominal pain. Additionally, he reported a 10 lbs weight loss over 1 year. There were no other gastrointestinal symptoms. Family history was noncontributory. Work up including complete blood count, electrolytes, creatinine and liver enzymes were normal.

**Figure 1.**
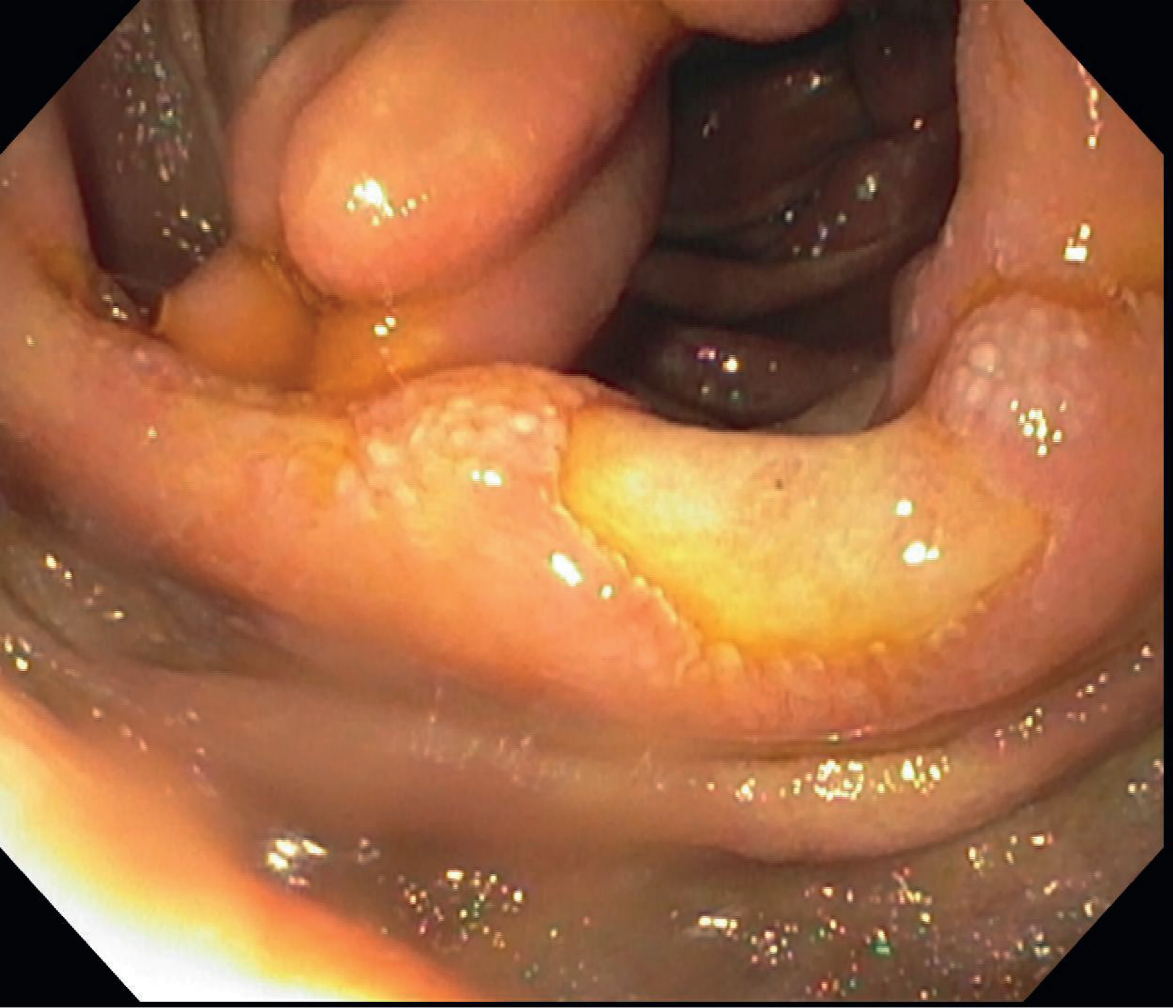
Significant deep ulcerated area noted in the jejunal fold.

**Figure 2.**
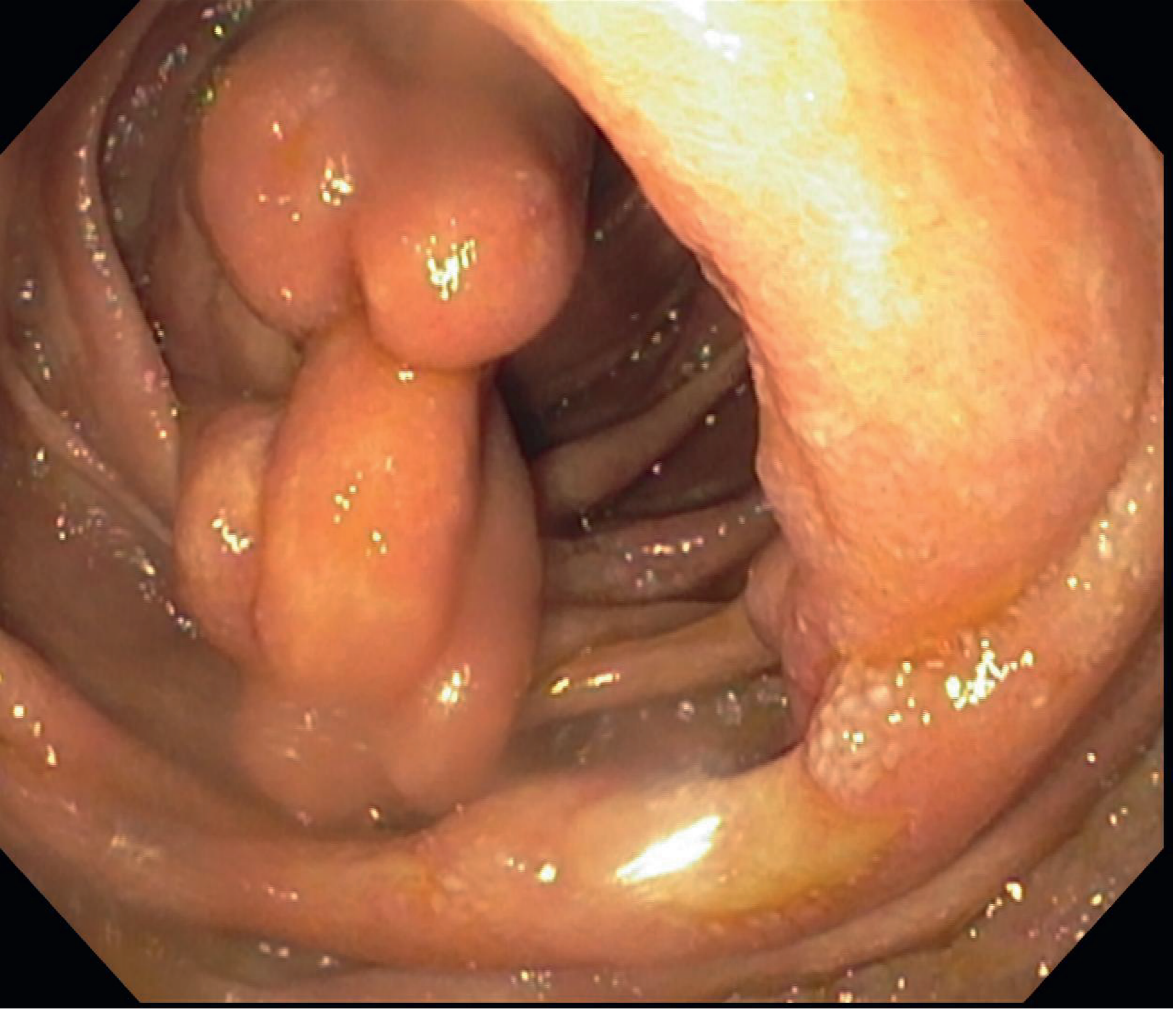
Multiple polypoid growth projection with normal overlying mucosa.

**Figure 3.**
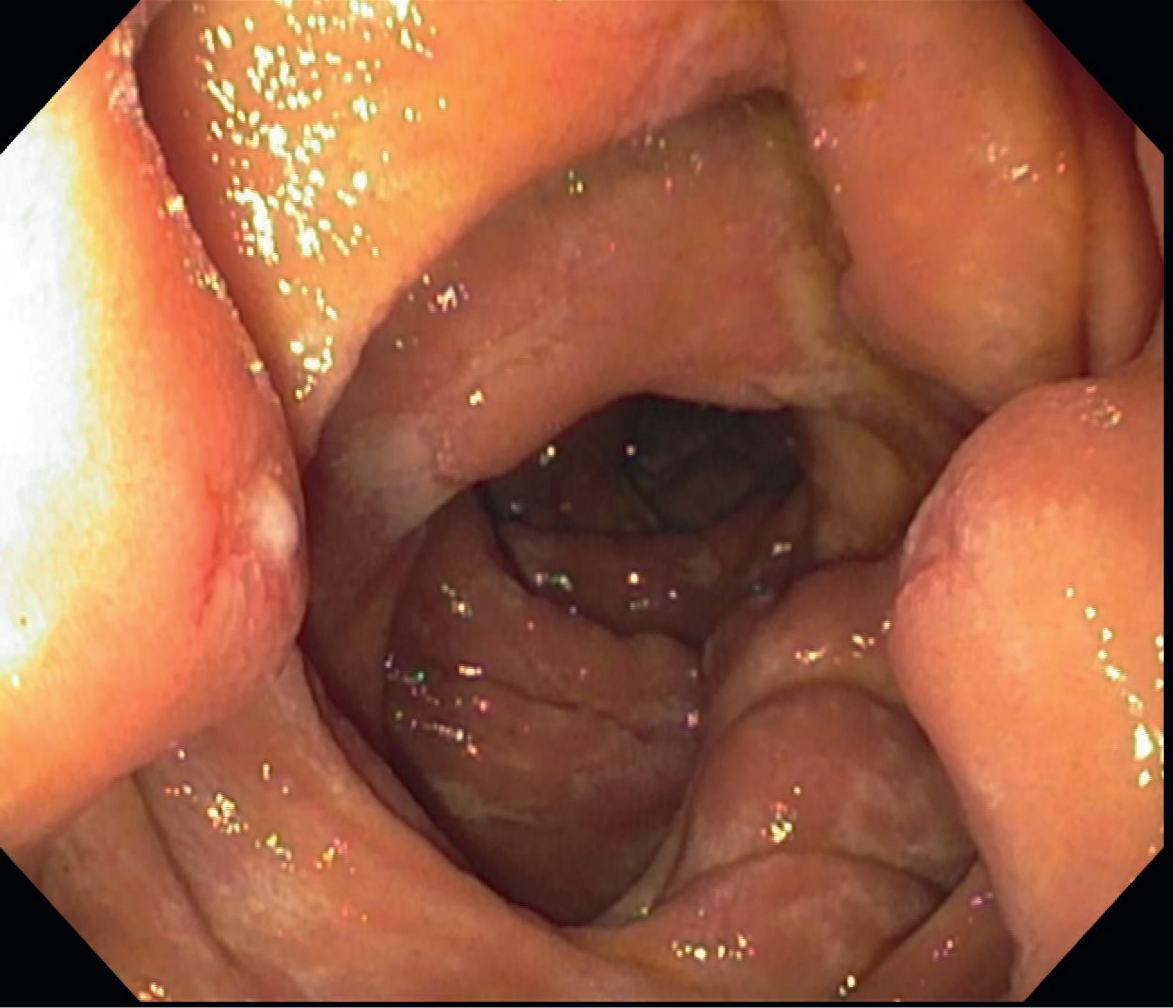
Widespread superficial ulcerations at the jejunum.

Double Balloon enteroscopy showed large ulcerated, friable and polypoid submucosal projections in the jejunum (Figures 1–3). Pathological examination of the biopsies demonstrated inflammation, plasma cells and positive congo red staining. Mass spectrometry performed on the biopsies was consistent with Lambda light chain (AL) amyloidosis.

Subsequently, a bone marrow biopsy was indefinite for plasma cell dyscrasia (<5% plasma cell with kappa predominance). Fluorescence in situ hybridization testing for t(11,14) was negative. Serology and urinary protein electrophoresis and a thorough cardiac and ophthalmologic assessment reveled no evidence of amyloidosis elsewhere. He was commenced on Cyclophosphamide-Bortezomib-Dexamethasone (CYBOR-D). Isolated small bowel amyloidosis is a rare disease entity and has been infrequently reported in the literature.

